# Impact of the Transcriptional Regulator SCO7424 Overexpression on Antibiotic Production in *Streptomyces coelicolor*

**DOI:** 10.3390/antibiotics15010070

**Published:** 2026-01-08

**Authors:** Gladys Vega-Sauceda, Karen Villarreal-Gómez, Beatriz Ruiz-Villafán, Romina Rodríguez-Sanoja, Sergio Sánchez

**Affiliations:** Instituto de Investigaciones Biomédicas, Universidad Nacional Autónoma de México (UNAM), Mexico City 04510, Mexico; glamarvega@comunidad.unam.mx (G.V.-S.);

**Keywords:** *Streptomyces coelicolor*, antibiotic production, carbon catabolite repression, transcriptional regulation, metabolic engineering

## Abstract

**Background.** The genus *Streptomyces* is known for its capability to produce a wide range of bioactive secondary metabolites. The enzymes required for their synthesis are encoded within biosynthetic gene clusters (BGCs), whose expression can be influenced by various physical and nutritional factors. Among these nutritional factors, it is worth highlighting carbon catabolic repression (CCR), which prevents the formation of secondary metabolites. It has been shown that transcriptional factors, in turn, regulated by glucose or by the enzyme glucose kinase (Glk), may be involved in this mechanism. It was shown that the expression of some transcriptional factors is regulated by glucose availability and that the enzyme glucose kinase (Glk) may play a role in this process. One of the transcriptional factors most upregulated in the presence of glucose/agar in *Streptomyces coelicolor* M145 is SCO7424, a member of the MarR family of transcriptional regulators. However, its influence on antibiotic synthesis has never been studied. **Objective.** In this work, we evaluated the effect of SCO7424 overexpression on the synthesis of actinorhodin (ACT) and undecylprodigiosin (RED), and its impact on growth and glucose consumption. **Methods.** A copy of the *sco7424* gene was cloned into the pIJ702 plasmid, which was then transformed into a wild-type strain of *S. coelicolor* M145. Growth and antibiotic production were evaluated in the strain with two copies of *sco7424* and in the wild-type strain. We also evaluated the expression of the probable target genes by quantitative RT-PCR. **Results**. We found that overexpression of *sco7424* negatively impacts growth, glucose consumption kinetics, and the expression of specific regulators of the ACT and RED biosynthetic pathways, resulting in reduced ACT and RED production. Understanding the function of the regulatory cascades regulated by this family of regulators is crucial for boosting the yields of valuable metabolites produced by industrial strains.

## 1. Introduction

The genus *Streptomyces* consists of Gram-positive, filamentous, aerobic bacteria commonly found in soil as saprophytes [1]. These bacteria are a significant source of secondary metabolites with antibacterial and antitumor properties [2].

The synthesis of these secondary metabolites is influenced by various nutritional factors, including carbon and nitrogen concentrations [3]. This implies the existence of multiple regulatory mechanisms, including carbon catabolite repression (CCR). CCR prioritizes carbon sources based on metabolic efficiency, favoring easily metabolized compounds for rapid growth. It downregulates genes associated with less advantageous sources, reducing metabolic costs and optimizing energy use, allowing the organism to maximize growth while conserving resources [4,5].

D-glucose is recognized as the preferred carbon source for optimal growth in *Streptomyces coelicolor* [6]. It represses genes involved in the use of alternative carbon sources [7]. Additionally, the production of secondary metabolites in *S. coelicolor* is suppressed by D-glucose [6,8].

Research has shown that glucose affects the expression levels of various transcriptional regulators [7]. This study identified at least 20 transcriptional regulators with differential expressions when comparing *S. coelicolor* M145 grown with glucose or agar as the carbon source. Notably, the *sco7424* gene exhibited the highest level of overexpression (log2 = 3.65) after 12 h of growth with glucose as the sole carbon source. This gene is located upstream of a Major Facilitator SCO7425 (MFS) and is probably co-transcribed with it. Based on its DNA sequence, *sco7424* belongs to the MarR family of transcriptional regulators, which includes proteins that play critical roles in antibiotic resistance, virulence, phenolic compound degradation, and osmoadaptation [9,10].

MarR proteins typically contain a winged helix-turn-helix motif that facilitates DNA binding [11]. Most of these proteins bind to AT-rich (pseudo)palindromic sequences in the intergenic regions between divergently oriented genes. This binding can inhibit gene expression by preventing RNA polymerase from attaching to the promoter. However, these regulators can also function as activators either by stabilizing RNA polymerase or by competing with other repressors for access to the promoter. Their structure is often responsive to ligands and includes a binding pocket for small molecules [12].

In the *Streptomyces* genus, some MarR regulators promote antibiotic production by acting as transcriptional repressors or activators of genes involved in antibiotic biosynthesis. For example, LcbR2 influences lincomycin synthesis in *Streptomyces lincolnensis* [13], DptR3 regulates daptomycin production in *Streptomyces roseosporus* [14], and SAV4189 is implicated in avermectin synthesis by *Streptomyces avermitilis* [15]. Conversely, some MarR regulators, such as CtcS in *Streptomyces aureofaciens*, repress antibiotic production, including tetracycline and chlortetracycline [16].

Currently, no information is available on the role of the putative MarR transcriptional regulator SCO7424 in growth and secondary metabolism in *S. coelicolor*. Herein, we investigated the effects of SCO7424 overexpression, a putative MarR-family member in *S. coelicolor*. The goal was to elucidate the regulatory role of SCO7424 in glucose-mediated CCR and to determine whether it influences the biosynthesis of actinorhodin (ACT) and undecylprodigiosin (RED) under glucose-repressive conditions. We hypothesize that SCO7424 acts as a central transcriptional regulator that links glucose availability to the control of antibiotic production in *S. coelicolor*.

## 2. Results

### 2.1. Three-Dimensional (3D) Model of the SCO7424 Controller

To understand the topological characteristics of this regulator and identify conserved residues that could indicate whether the protein product is functional, a three-dimensional model of the protein was generated using Phyre2. Phyre2 was fed with the MarR protein A0A4S2WEG8_9ACTN from *Streptomyces* sp. LRa12 as a template.

**Figure 1:**
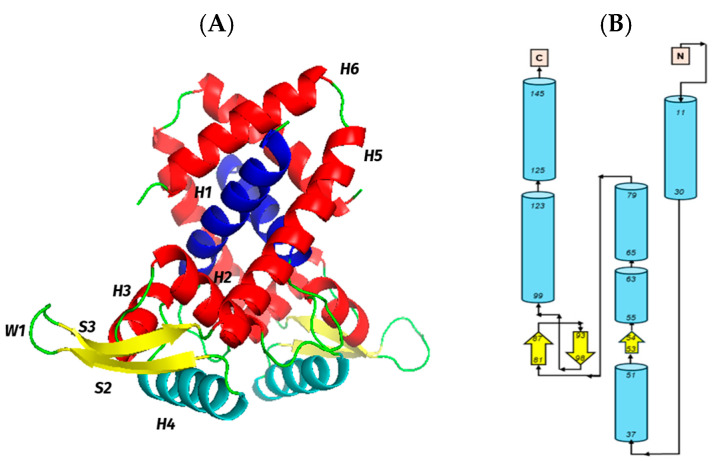
(**A**) Putative 3D model obtained by Phyre2 V.2 of the MarR transcriptional regulator homodimer SCO7424. Shown in yellow are the two strands that form the wing of each of the monomers. In red, the 6 alpha-helices (H1–H6) are observed. The helix (H4) is marked in cyan, where the binding domain to the major groove of DNA is located. Marked in navy blue, the helixes (H1) corresponding to the dimerization domain are highlighted. (**B**) Topology of SCO7424 was obtained using Jpred 4, in blue the amino acids forming the helices, and in yellow the ones forming the strands.

As shown in Figure 1, the SCO7424 regulator consists of six alpha-helices, three strands, and loops/twists per monomer, arranged as follows: H1-H2-S1-H3-H4-S2-W1-S3-H5-H6.

**Figure 2:**
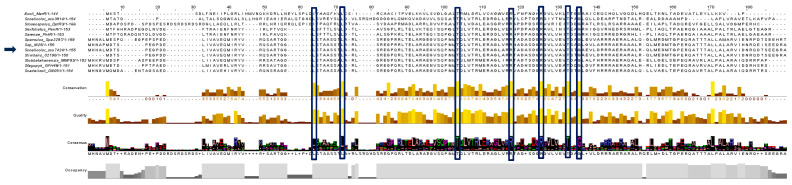
Multiple alignment of SCO7424 and 11 orthologous sequences. Highlighted in bars are the conserved amino acids.

Likewise, in the alpha helix corresponding to the DNA-binding domain, it is possible to appreciate the presence of conserved amino acids such as T70 and R84 (Figure 2). The amino acids R92, T99, and G102 are also conserved in a region corresponding to the beginning of the second strand of the wing and the continuous alpha helix. Simultaneously, the presence of two highly conserved leucines (L36 and L44) is observed in helix 2 (H2), and the absence of cysteines in the structure is noted.

In the multiple alignment, it can be observed that the amino acid regions where the ligand- and DNA-binding domains are located show a high degree of similarity to those of the PntR and PenR regulators. This suggests that the SCO7424 ligand molecule may share chemical and/or structural characteristics with pentalenolactone, the effector molecule of the previously mentioned regulators [17].

**Figure 3:**
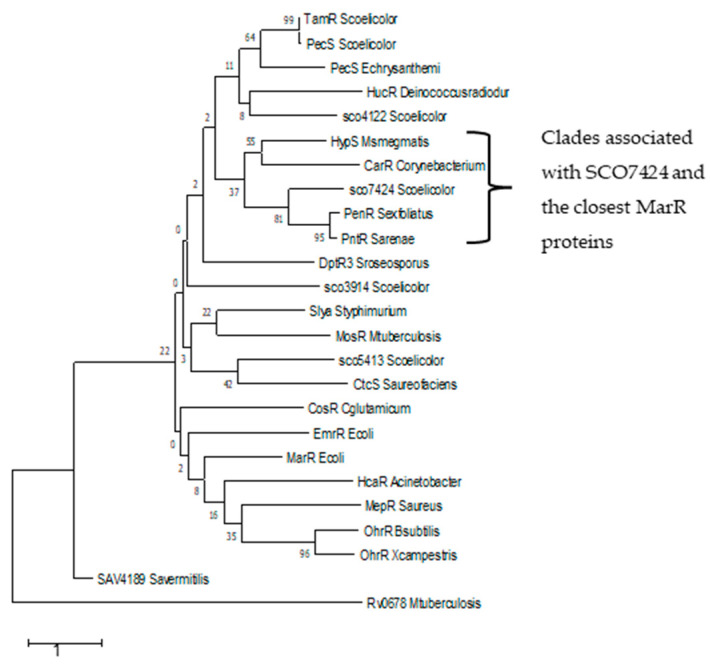
Phylogenetic tree of SCO7424 and 24 orthologous sequences. MarR *Escherichia coli* (AAK21292.1), HcaR *Acinetobacter baylyi* ADP1 (CAG68570.1), EmrR *E. coli* (WP_159385333.1), TamR *S. coelicolor* (WMT33768.1), PecS *S. coelicolor* (CAB95926.1), PecS *Erwinia chrysanthemi* (AAF74777.1), HucR *Deinococcus radiodurans* (PDB:2FBK_B), Rv0678 *Mycobacterium tuberculosis* (CAI9304756.1), Slya *Salmonella enterica* subsp. *Enterica serovar* Typhi (BAA24582.1), MosR *M. tuberculosis* CDC1551 (AAK45331.1), MepR *Staphylococcus aureus* (AAU95767.1), OhrR *Bacillus subtilis* (WP_326353889.1), OhrR *Xanthamonas campestris* (PDB: 2PEX), CosR *Corynebacterium glutamicum* (GAV98202.1), HypS *Mycobacterium smegmatis* MC2 155 (ABK72338.1), SAV4189 *Streptomyces avermitilis* (BAC71901.1), PenR *Streptomyces exfoliatus* (ADO85585.1), PntR *Streptomyces arenae* (ADO85569.1), SCO5413 S. *coelicolor* (CAB70643.1), SCO3914 *S. coelicolor* (CAB42742.1), DptR3 *Streptomyces roseosporus.* (AAX31530.1), CtcS *Streptomyces aureofaciens* (AEI98662.1), CarR *C. glutamicum* (WBG74556.1), SCO4122 *S. coelicolor* (CAB92375.1), SCO7424 *S. coelicolor* (CAB76343.1). Evolutionary history was inferred using the maximum-likelihood method with the Le and Gascuel model [18]. Initial trees were created using the Neighbor-Joining method based on JTT model distances. A discrete gamma distribution was used to model differences in rates between sites. Analyses were conducted in MEGA X [19] with 1000 bootstrap replicates.

Based on this, a phylogenetic tree of the MarR regulators was constructed (Figure 3). This tree shows that SCO7424 is grouped not only with PntR and PenR, but also with HypS and CarR, regulators belonging to other actinobacteria that can respond to antibiotics.

### 2.2. Determination of the Putative Promoter Region of SCO7424

**Figure 4:**
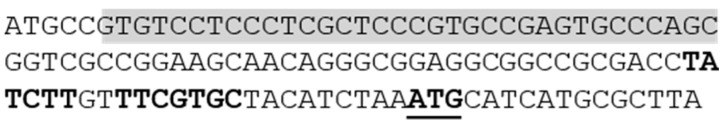
Putative promoter site of *sco7424*. Sequences of the putative promoter of *sco7424* are in bold, the start codon is in bold and underlined. The putative operator site of the SCO7424 regulator is highlighted in gray.

To identify the putative promoter region of SCO7424, we first attempted to locate the canonical hexamer of the *Streptomyces* ribosome binding site (AGGAGG) located just upstream of the ATG start codon (Figure 4). This attempt was unsuccessful, suggesting that the transcriptional and translational sites overlapped. Thus, we searched for putative −10 and −35 sequences just upstream of the ATG codon. Putative, rather than canonical *Streptomyces* −10 (TANNNT) and −35 (TTGNCN) sequences were identified [20,21]. However, these findings should be validated using S1 mapping or RNA sequencing.

### 2.3. sco7424 and sco7425 Are Co-Transcribed from a Promoter Located Upstream of sco7424

**Figure 5:**
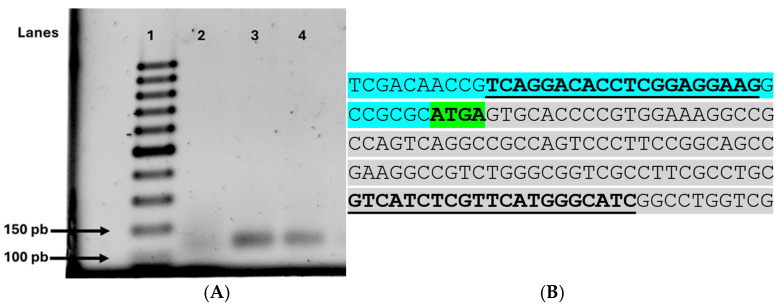
(**A**). Agarose gel electrophoresis (1.2%) of *sco7424* and *sco7425*. Lane 1: GeneRuler 100 bp DNA Ladder weight marker. Lane 2: Negative control (RNA without reverse transcription). Lane 3: Positive control (genomic DNA). Lane 4: 24 h agar cDNA. (**B**). DNA sequence of the primers used for the RT-PCR assay to determine the co-transcription of genes *sco7424* and *sco7425*. The sequence of *sco7424* is in blue, and the sequence of *sco7425* is in gray. The TGA codon of *sco7424* and the ATG codon of *sco7425* overlap and are marked in green and bold. The forward and reverse primers for RT-PCR are underlined and in bold.

A bioinformatic analysis using the Operon Mapper program was conducted to determine whether *sco7424* and *sco2425* genes form an operon. The analysis predicted that both genes are co-transcribed from a promoter located upstream of *sco7424* (Figure S1). This prediction was experimentally confirmed by a PCR assay using cDNA derived from total RNA of the WT strain *S. coelicolor* M145. The results showed that both genes were cotranscribed when the strain was grown with 0.5% agar and 0.5% glucose as carbon sources (Figure 5, lane 4).

In addition, RT-PCR analysis of a 135 bp region encompassing both genes, including the overlapping region, and sequencing the resulting product (Figure S1) further supported that the expression of these two genes is likely driven by the same promoter located upstream of *sco7424.*

### 2.4. Determination of the Putative SCO7424 Target Genes

To predict the binding site and potential target genes of this regulator, we first obtained sequences spanning −400 bp to +50 bp for 40 orthologs of sco7424. This approach is based on the premise that the regulator can self-regulate; therefore, its promoter—and, consequently, the binding site—must be within the specified range of base pairs [22,23]. This latter was used to search for orthologous sequences in other members of the Streptomyces genus. We selected 40 of these sequences to create the input database for the MEME tool. Of the five signatures obtained, we selected the one with the best E-value (<10) and *p*-value (<0.001) for the subsequent prediction of SCO7424 regulator target genes (Figure S1). This sequence does not exhibit palindromicity. It is in the intergenic region between *sco7424* and *sco7423,* which aligns with the generality reported for regulators of this family [12].

**Figure 6:**
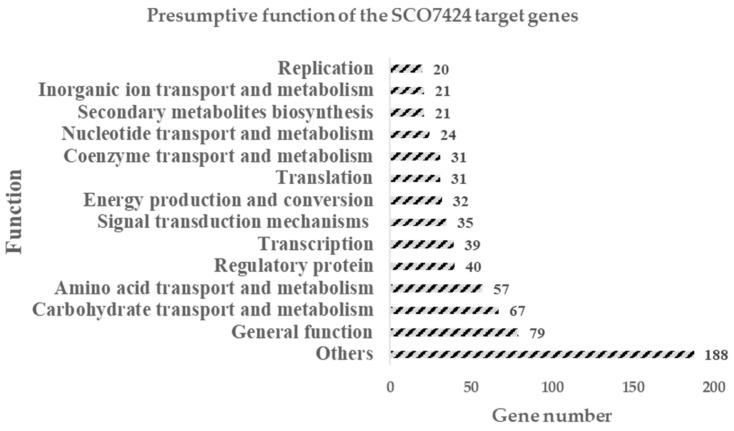
Graph of putative target genes for SCO7424 obtained by MAST and their presumptive functions. Other functions include genes with unknown functions, the mobilome, and more.

An analysis of the signature with the MAST program showed that the transcriptional regulator SCO7424 has 685 potential gene targets, of which 21 are involved in the synthesis of secondary metabolites, 40 in regulatory processes, and 35 in signal transduction in *S. coelicolor* (Figure 6). Among the 21 genes involved in secondary metabolism, several encode enzymes implicated in the synthesis of ACT, RED, and CPK, a type I polyketide. For ACT, the genes *sco5083 (actII-2)* and *sco5091 (actIV)* were detected. These genes code for a putative transport protein and a cyclase, respectively. In the case of RED, the genes *sco5898 (redF*) and *sco5880 (redY*) were identified, encoding an oxidoreductase and a protein of unknown function, respectively. Additionally, the gene *sco6284* (*cpkK*) was identified, encoding a decarboxylase.

Similarly, it is possible to observe target genes influencing carbon metabolism (67 genes) and amino acid metabolism (57 genes), representing the target gene groups with the highest percentages (9.7% and 8.3%, respectively).

### 2.5. Characterization of the SCO7424 Overexpressed Mutant (OVE) Strain pIJ702/sco7424

#### 2.5.1. Growth and Glucose Consumption

**Figure 7:**
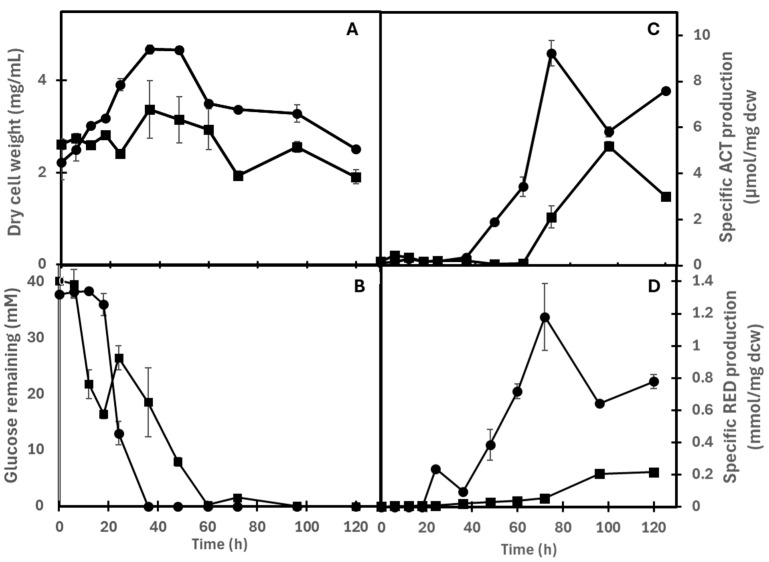
(**A**) Evolution of the dry weight of the M145 (WT) strain (●) and the *sco7424* OVE strain (■) grown in NMMP medium supplemented with glucose and agar (0.5% *w*/*v* each). (**B**) Glucose remained throughout fermentation. Time points marked with an asterisk indicate a significant difference (*p* < 0.05). (**C**) Production of actinorhodin and (**D**) undecylprodigiosin from the wild-type (WT) (●) and OVE strains (■).

As shown in Figure 7A and Table 1, the WT and OVE strains reached their maximal biomass yield after 36 h of fermentation. After this point, biomass decreased, suggesting a lytic process in both strains. In the WT strain, an abrupt decrease in glucose concentration in the growth medium was observed only after 20 h of cultivation and continued steadily from 20 to 40 h, correlating with active growth of the strain. These observations suggested that the strain used carbon storage compounds to initiate and sustain growth before 20 h. The WT strain reached glucose exhaustion after 36 h of cultivation (Figure 7B).

In contrast, the pattern of the decrease in glucose concentration in the growth medium of the OVE strain was quite different and more complex than that of the WT strain. Initially, glucose concentration decreased rapidly as early as eight h after the start of cultivation, reaching a minimum at 20 h (Figure 7B). A rapid, early growth phase did not accompany this abrupt uptake and is thus likely to result in a high intracellular glucose concentration. Surprisingly, between 20 and 24 h, the glucose concentration in the growth medium increased, suggesting the release of intracellular glucose. This phase coincided with a period of slow growth, followed by a gradual decrease in glucose concentration between 24 h and 60 h, during which active growth occurred (Figure 7A). The OVE strain reached glucose exhaustion 24 h later than the WT strain, at 60 h of cultivation. Overall, the wild-type strain M-145 exhibited faster growth and better biomass yield than the OVE strain. The reduced growth of the OVE strain correlated with lower glucose consumption, which may be either the cause or the consequence of the lower growth rate. Both strains achieved their maximal biomass yield after 36 h of fermentation. After this point, biomass decreased, suggesting the onset of lysis in both strains (Figure 7A,B and Table 1).

#### 2.5.2. Production of ACT and RED

As shown in Figure 7C,D, production of ACT and RED in the WT strain started when glucose was depleted from the growth medium (around 36 h) during the stationary phase, and peaked at 72 h. In the OVE strain, ACT and RED also began after glucose was exhausted in the growth medium (Table 1). Still, since glucose was exhausted from the growth medium 24 h later in the OVE strain than in the WT strain (around 60 h), ACT and RED production peaked at 96 h rather than at 72 h and were two-fold and five-fold lower, respectively, in this strain than in the WT strain. These results indicate that the onset of ACT and RED production occurs when glucose is depleted from the growth medium. Because glucose exhaustion was delayed by 24 h in the OVE strain, ACT and RED production were also delayed and lower than in the WT strain. These results suggest that SCO7424 overexpression likely indirectly represses expression of the RED and ACT clusters.

#### 2.5.3. Impact of sco7424 Overexpression on Its Transcript Level and on That of sco7423 and sco7425

As previously reported, regulators in the MarR family usually control their own expression and that of genes divergently oriented [24], so the transcript levels of sco7424 and sco7423 were compared in the WT and OVE strains. sco7423 encodes a protein of unknown function bearing an ANTAR-GAF domain. This domain can function as an anti-terminator by binding to and stabilizing the dual hexaloops of RNA motifs in the nascent RNA strand during transcription, thereby preventing the formation of a terminator loop [25,26]. These analyses were conducted with mRNA prepared during the exponential phase (at 24 h for WT and 36 h for OVE, when glucose is present in the growth medium) and in the stationary phase (at 48 h for WT and 72 h for OVE, after glucose exhaustion). qRT-PCR analyses indicated that the *sco7424* overexpression profile was maintained in the OVE strain under conditions of glucose availability and depletion (Table 2). Interestingly, *sco7423* expression was higher in the OVE strain than in the WT strain, at least when glucose was present in the growth medium, but decreased after glucose exhaustion. This suggested that the expression of *sco7423* might be under the positive control of *sco7424*, but that other regulatory factors are also involved in its regulation.

#### 2.5.4. Impact of sco7424 Overexpression on Transcript Level of Regulatory Genes Belonging to the RED (*redD*, *redL*, *redZ*) and ACT (*actII-ORF4*) Clusters

The expression levels of the cluster-specific regulators of RED (*redD*, *redZ*, *redL*) and ACT (*actII-ORF4*) were also compared between the WT and OVE strains. These analyses were conducted with mRNA prepared under the same conditions as mentioned above. In line with the suppressive effect of SCO7424 on ACT and RED production, the *redD*, *redL*, and *actII-ORF4* transcripts were present at lower levels in the OVE compared to the WT strain in the presence of glucose and at even lower levels after glucose exhaustion (Table 2).

Changes in gene expression were calculated using the normalized expression values relative to the reference gene *hrdB,* a constitutive gene encoding the principal sigma factor.

## 3. Discussion

Previously, our research group conducted a transcriptional analysis of the *S. coelicolor* M-145 strain under glucose repression conditions. We found that at least 20 transcriptional regulators were differentially expressed in the presence of glucose, compared to non-repressive conditions on agar [7]. Among these, the *sco7424* gene, believed to encode a MarR-family transcriptional regulator, was the most overexpressed, with a log2 value of 3.65 after 12 h of growth compared to agar.

To gain insight into the probable function of SCO7424, a three-dimensional model was generated, confirming that it has a typical MarR transcriptional factor topology. This regulator harbors conserved amino acids within its DNA-binding domain, which are considered potential ligand-binding sites, providing insight into the chemical nature of the effector molecules likely to drive conformational changes in this regulator. These amino acids (T70, R84, R92, T99, and G102) have been shown to interact with molecules such as salicylate and other aromatic compounds, suggesting that SCO7424 might respond to a metabolite of this type [22]. On the other hand, the absence of cysteines in its structure suggests that this protein is not responsive to oxidative stress [11]. Therefore, the protein encoded by *sco7424* possesses the necessary features to function as a MarR transcriptional factor and may respond to molecules other than ROS [12].

Studies using Operon Mapper, PCR analysis of cDNA samples, and sequencing of the amplification products indicate that *sco7424* and *sco7425* form an operon and are co-transcribed from a promoter upstream of *sco7424* (Figure 5) [15,27,28,29,30]. *sco7425* encodes a putative multi-drug-efflux transporter protein, whereas *sco7423*, which is present in divergence of *Sco7424*, encodes a protein of unknown function bearing an ANTAR-GAF domain and thus might function as an anti-terminator [25,26].

Concerning the putative promoter sites, the detection of the SCO7424 signature provides evidence for self-regulatory control over the levels of the corresponding MarR regulator product and its adjacent *sco7425* gene.

Regarding the presumptive regulator target genes associated with SCO7424, a non-palindromic signature was used. This sequence was also identified in the intergenic region of *sco7424*, near the divergently oriented gene *sco7423*, which aligns with this family of regulators [12]. Among the target genes identified for this regulator, 21 are involved in secondary metabolite synthesis, including those encoding enzymes responsible for producing ACT, RED, and CPK. Additionally, target genes related to carbon and amino acid metabolism were identified. These include genes encoding sugar transporters and some involved in glucose metabolism, supporting the broad relevance of SCO7424 in cellular metabolism and antibiotic regulation.

The OVE strain exhibits a lower growth rate and a lower biomass yield than the WT strain. Interestingly, glucose uptake started earlier and was more active in the OVE strain than in the WT strain from 8 to 20 h (phase 1) but was not accompanied by active growth. This suggested that overexpression of *sco7424* stimulates glucose uptake, leading to a high intracellular glucose concentration, which is known to inhibit the growth in *Streptomyces* species [31]. Subsequently, an increase in glucose concentration in the growth medium was observed between 20 and 24 h (phase 2), suggesting the expulsion of excess glucose from the cell. After this phase 2, a steady decrease in glucose concentration in the growth medium was observed from 24 h to 60 h (phase 3), accompanied by a phase of active growth. However, it is noteworthy that glucose exhaustion from the growth medium occurred at 60 h in the OVE strain, 24 h later than in the WT strain.

Our results instead suggested that SCO7424 initially had a strong positive impact on glucose uptake. Still, this early, very active glucose uptake ultimately harmed the growth rate of the OVE strain, which might account for its lower glucose uptake. This condition was correlated with delayed and lower antibiotic production.

To explain our observations, we propose that in the condition of *sco7424* overexpression, the MFS encoded by *sco7425* might be overexpressed and promote glucose uptake. Alternatively, SCO7424 might positively regulate the expression of the genes encoding the two glucose permeases, which are thought to be the main proteins involved in glucose uptake [32]. Both hypotheses can explain the stimulatory effect of SCO7424 overexpression on glucose uptake from 8 to 20 h. This stimulation would result in a high intracellular glucose concentration, which, beyond a specific threshold, would be inhibitory for growth [31]. Consequently, an expulsion of excess glucose from the cell would be triggered to allow growth between 24 h and 60 h.

These hypotheses should be validated by deleting *sco7424*/or by deleting and overexpressing *sco7425* and *sco7423*, and by studying the impact of these manipulations on growth, glucose uptake, and antibiotic production as well as on the expression of GlcP and *sco7425*.

Interestingly, the WT and OVE strains both start producing ACT and RED after glucose exhaustion, but exhaustion occurs at 36 h in the WT strain and 24 h later, at 60 h in the OVE strain. The OVE strain shows significantly delayed and two and 5-fold lower production of ACT and RED, respectively, than the WT strain. These findings align with the qRT-PCR analysis, which indicated reduced expression levels of specific regulatory genes in the RED (*redL* and *redD*) and ACT (actII-ORF4) clusters in the presence of glucose and after glucose exhaustion.

Our data thus indicate that overexpression of *sco7424* harms growth, alters glucose consumption kinetics, and reduces the expression of specific regulators of the ACT and RED pathways, thereby reducing the production of ACT and RED.

**Figure 8:**
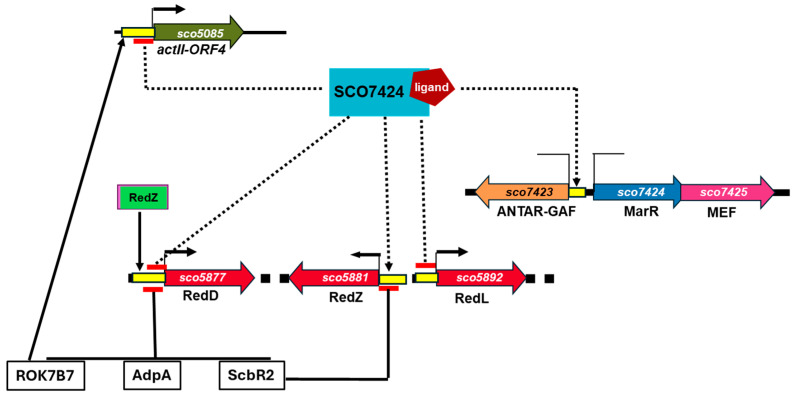
Proposed regulatory model for the MarR SCO7424 regulator. Dotted arrows show the regulations mediated by SCO7424, either directly or indirectly (yet to be elucidated). Solid arrows show regulatory pathways previously reported in the literature.

The involvement of MarR family regulators in the regulation of antibiotic biosynthesis in the genus *Streptomyces* is well documented [22]; however, their potential to boost antibiotic production has not widely exploited. While MarR regulators are established as pivotal players in antibiotic production, their full potential remains locked. Our results demonstrate that the MarR protein SCO7424 negatively affects growth, glucose uptake, and secondary metabolite production (Figure 8); conversely, its disruption could be expected to have a positive effect on these processes. Disrupting *sco7424* and overexpressing or deleting *sco7425* or *sco7423* would be interesting to unravel the functions of these genes, assess their impact on growth, glucose uptake, and antibiotic production, and define strategies for genetic engineering to boost yields of valuable metabolites produced by industrial strains.

## 4. Materials and Methods

### 4.1. Strains, Plasmids, and Growth Conditions

*Streptomyces coelicolor* M145 was used as a wild-type (WT) strain to generate the SCO7424 overexpressed mutant (OVE). The WT strain and its derived OVE mutant are available at the UNAM-48/WFCC (Mexico City). The *Streptomyces* strains were stored as spore suspensions in 20% (*v*/*v*) glycerol at −30 °C as previously described [33]. The minimal liquid medium (NMMP) made up of [(NH_4_)_2_SO_4_ 0.2%, casamino acids 0.5%, MgSO_4_·7H_2_O 0.06%, PEG 6000 (5%)], supplemented with minor elements [33] was used to grow the *Streptomyces* strains; glucose and agar were added at a final concentration of 0.5%. Mannitol Soy Flour Agar (MS) medium (mannitol 2%, soy flour 2%, agar 2%, MgCl_2_ 10 mM) was used for sporulation of the strains. For protoplast recovery, R2 medium plates (containing 10.3% sucrose, 0.025% K_2_SO_4_, 1.012% MgCl_2_·6H_2_O, 1% glucose, 0.01% casamino acids, and 0.01% agar) were used, supplemented as described by Kieser et al. [34]. All strains and plasmids used and generated in this work are summarized in Table S1. Primers previously reported in other works and those designed in this study are listed in Table S2.

For generating the overexpressed strain, the high-copy-number plasmid pIJ702 was selected, purified, and digested with the restriction enzymes *Sac*I *and Bgl*II. For amplification of the *sco7424* gene, primers were designed to include plasmid restriction sites. Gradient PCR was performed to determine the optimal Tm for amplification using the Thermo Fisher Scientific (Waltham, MA, USA) Platinum Taq DNA Polymerase enzyme according to the manufacturer’s instructions. The linearized plasmid with *SacI* and *Bgl*II digestion, as well as the amplified and digested fragments of the *sco7424* gene, were purified from agarose with MP Biomedicals’ (Irvine, CA, USA) GeneClean kit. Ligation was performed using Thermo Fisher Scientific (USA) T4 DNA Ligase enzyme according to the manufacturer’s directions. The transformation was carried out in the *S. coelicolor* strain M145 using the protoplast formation protocol for *Streptomyces* transformation [34]. It is worth mentioning that, as a control, construction and transformation were carried out with the empty pIJ702 plasmid.

The presence of the plasmid in the colonies obtained was detected by colony PCR. The positive colonies were likewise propagated for the purification of the pIJ702 plasmid with the *sco7424* insert using the miniprep technique by alkaline lysis [35]. Double digestion was performed with the restriction enzymes *Sac*I and *Bgl*II, and the digestion products were visualized by electrophoresis on a 0.8% agarose gel. Once the expected product corresponding to the size of the *sco7424* gene was observed, two independent digestions were performed to verify that the gene was inserted in the correct direction. One digestion was performed with the restriction enzymes *Bgl*II and *Xho*I, and the other with *Sac*I and *Xho*I. Finally, to validate that the insert was the *sco7424* gene, a PCR was executed using the PIJ702 oligonucleotides (Table S2).

The PCR product obtained was purified using Promega’s Wizard^®^ (Madison, WI, USA) SV Gel and PCR Clean-Up kit and sent for sequencing. The obtained sequence of pIJ702/*sco7424* in the transformed strain showed 100% identity with the sco7424 gene in the StrepDB database (https://strepdb.streptomyces.org.uk/ (accessed on 13 November 2023)), indicating the absence of mutations and its correct location within the reading frame.

### 4.2. 3D Model of the SCO7424 Regulator

The amino acid sequence of SCO7424 from *S. coelicolor* was obtained from StrepDB (https://strepdb.streptomyces.org.uk/ (accessed on 20 November 2023)). To obtain the three-dimensional structure of the SCO7424 regulator, the bioinformatic tool Phyre2 (http://www.sbg.bio.ic.ac.uk/phyre2/html/page.cgi?id=index (accessed on 23 November 2023)) was used, namely the PDB file from the AlphaFold Protein Structure Database (https://alphafold.ebi.ac.uk/ (accessed on 27 November 2023)) for the A0A4S2WEG8_9ACTN protein, corresponding to a transcriptional regulator of the MarR family in *Streptomyces* sp. LRa12, was utilized as a template. The mode used was “expert”, as well as the “One-to-one threading” tool, maintaining the default parameters [36]. The PDB file generated by the program was used as input in PyMOL version 2.5 (https://pymol.informer.com/2.5/ (accessed on 27 November 2023)) to facilitate the study of each secondary structure of this controller. The prediction of the secondary structures was obtained using JPred 4 (https://www.compbio.dundee.ac.uk/jpred4/index.html (accessed on 27 November 2023)) and PDBsum (https://www.ebi.ac.uk/thornton-srv/databases/pdbsum/ (accessed on 29 November 2023)).

### 4.3. Prediction of the DNA Binding Site of SCO7424 and Potential Target Genes

To carry out the prediction of the binding site and the possible target genes of this regulator, the sequences of −400 bp and +50 of 40 orthologous genes to the gene of the regulator *sco7424* were obtained in the first instance, since it is based on the scoop of the ability of the regulator to self-regulate, so its promoter, and, therefore, the binding site, must be in the area of the previously mentioned base pairs [7,22]. We selected orthologs using KEGG, a curated database that provides greater annotation reliability than standard BLAST (https://blast.ncbi.nlm.nih.gov/Blast.cgi (accessed on 3 August 2024)) searches, which often include poorly annotated sequences. Our criteria for ortholog selection were a minimum of 70% overall coverage and at least 30% sequence identity, not similarity.

The sequences were obtained from the KEGG database (https://www.genome.jp/kegg/ (accessed on 14 August 2024)), selecting those with the best BRH (Best Reciprocal Hit) value, and their descriptions were not labeled as “putative”. Our criteria for orthologs selection were a minimum of 70% overall coverage and at least 30% sequence identity. Subsequently, these sequences were formatted in multiple FASTA formats and used as input to the MEME program (https://meme-suite.org/meme/tools/meme (accessed on 22 October 2024)) to predict consensus sequences (possible junction sites of the regulator). The search was limited to motifs or binding sites with a maximum length of 35 nucleotides, as this is the average binding-site length for transcriptional factors in the genus *Streptomyces* [37].

The program was asked to produce at least five consensus sequences, both palindromic and non-palindromic. Then, each motif was evaluated using MAST (https://meme-suite.org/meme/tools/mast (accessed on 16 October 2024)) against a database containing the −400 and +50 sequences of all *S. coelicolor* A3 genes. The alignments were considered significant if they had an expectancy value (E-value) of less than 10 and a positional value less than 0.0001, as these values ensure a more probable binding site. Finally, the motif with the highest number of target genes was chosen [38].

### 4.4. In Silico Determination of the Promoter Zone of the SCO7424 Regulator

The search for possible promoter sequences was carried out using the G4PromFinder package (https://github.com/MarcoDiSalvo90/G4PromFinder (accessed on 14 August 2024)), using the complete genome of *Streptomyces coelicolor* A3(2) (NC_003888.3) as input information. This program identified potential promoter zones based on the presence of 25 bp “−10” sequences rich in TA, with approximately 40% TA content. Subsequently, once this area was detected, the second step consisted of identifying the putative G-quadruplex motifs extended up to 50 bp upstream of the 5′ end of the TA-rich sequence [39].

The output information obtained was the coordinates of the possible promoters in the genome. Based on this information, the putative promoter zones were selected at −400 bp and +50 bp relative to the sco7424 gene. Once these zones were selected, they were used as input to determine whether the regulator junction consensus sequence was SCO7424 in any of them.

### 4.5. Operon of the sco7424 and sco7425 Genes

Because the *sco7425* gene overlaps the *sco7424* gene, it was inferred to be transcribed as part of a polycistronic transcript, likely forming an operon. To confirm this theory, possible operons were predicted using the Operon Mapper (https://biocomputo.ibt.unam.mx/operon_mapper/ (accessed on 16 August 2024)) tool with the default configuration [40]. Then, based on the results, oligos were designed (Table S2) to amplify both genes from cDNA, enabling determination of whether they are co-transcribed. The resulting amplification product was purified using Promega’s Wizard^®^ (USA) SV Gel and PCR Clean-Up System kit and finally sent for sequencing. The sequences of oligonucleotides that amplify the genes *sco7424* and *sco7425* are shown in Table S2.

### 4.6. Determination of Bacterial Growth and Glucose Consumption

To investigate growth kinetics, 1 × 10^8^ spores of each *S. coelicolor* strain were inoculated in baffled flasks containing 50 mL of NMMP medium supplemented with the respective carbon sources: 0.5% glucose and 0.5% agar (repressive condition). Subsequently, they were incubated at 29 °C while stirring at 200 rpm for 120 h, taking samples of the medium at various times during fermentation.

To determine growth using dry weight, 5 mL of medium was filtered through a Whatman No. 1 filter and dried at 60 °C for 48 h. Residual glucose was quantified with the Trinder method of Spinreact Reagent (Girona, Spain) [8].

### 4.7. Quantification of ACT and RED

Antibiotic quantification was performed by spectrophotometry. For the ACT method, 0.5 mL of 3 N KOH was added to 0.5 mL of culture, and the treated samples were incubated at room temperature overnight (16–18 h) with agitation. Subsequently, the samples were centrifuged at 7000 rpm for 10 min, and the absorbance of the supernatant was measured at 640 nm. Finally, a molar extinction coefficient of 25,320 M^−1^ cm^−1^ was used to determine the concentration of ACT [5].

RED was extracted from 1 mL of culture medium containing dried mycelium, which had been previously centrifuged at 7000 rpm for 10 min. 1 mL of MeOH acidified with 0.5 M HCl was added to the mycelium obtained, and the samples were incubated at 4 °C overnight (16–18 h) in a stirrer. Finally, the samples were centrifuged at 7000 rpm for 10 min, and their concentration was determined by measuring the absorbance of the supernatant at 530 nm, using a molar extinction coefficient of 100,500 M^−1^ cm^−1^ [5].

### 4.8. RNA Isolation

RNA isolation was performed from NMMP cultures supplemented with glucose (0.5%) and agar (0.5%), inoculated with 1 × 10^8^ spores of the wild strain (*S. coelicolor* M145) and the over-expressing strain pIIJ702/sco7424, respectively. Samples were taken under two metabolic conditions: (1) even in the presence of glucose, at 24 h (M-145) and 36 h (OVE) fermentation, and (2) in the absence of glucose, at 48 h (M-145) and 72 h (OVE) incubation.

The mycelium recovered after centrifugation at 7000 rpm for 10 min was stabilized with Qiagen’s RNAprotect reagent (Venlo, The Netherlands) according to the manufacturer’s instructions. The RNA was then isolated using the Qiagen RNeasy Mini Kit (The Netherlands) according to the manufacturer’s instructions. Likewise, RNA integrity was verified by 1% agarose gel [8].

### 4.9. RT-qPCR

cDNA was synthesized using SuperScript^®^ III Reverse Transcriptase (Invitrogen, Grand Island, NY, USA), following the manufacturer’s instructions. To obtain it, RNA quantities ranging from 10 pg to 5 μg were used, along with random hexamers. Dilutions of the obtained cDNA were used as a template to perform the RT-qPCR assays and the standard/melting curves (160 ng/μL, 80 ng/μL, 40 ng/μL, 20 ng/μL, 10 ng/μL, five ng/μL, and 2.5 ng/μL).

For qPCR assays, 10 μL reactions containing one μL of dilute cDNA (20 ng), one μL of each set of primers (5 μM) with a final concentration of 0.5 μM, five μL of Maxima SYBR Green/ROX qPCR Master Mix 2X (Thermo Fisher Scientific, USA), and three μL H_2_O [8,41]. The assay runs were performed on a Bio-Rad’s CFX96 Touch Real-Time PCR Detection System (96-well white plates, Hercules, CA, USA), using a 10 min cycle for initial denaturation at 95 °C, and 40 cycles of 0.15 min for denaturation at 95 °C and 1.0 min at 60 °C for alignment/extension.

Changes in gene expression were calculated using the normalized expression values relative to the reference gene *hrdB*, a constitutive gene encoding the principal sigma factor. The formula used by the CFX Maestro software #12004110 (https://www.bio-rad.com/es-mx/sku/12004110-cfx-maestro-software?ID=12004110 (accessed on 4 June 2025) to calculate normalized expression is as follows (Bio-Rad Laboratories, Hercules, CA, USA):

$$\mathrm{Normalized}\;\mathrm{expression}=\frac{RQ_{\mathrm{sample}\;(\mathrm{target}\;\mathrm{gene})}}{{\left(RQ_{\mathrm{sample}\;(\mathrm{reference}\;\mathrm{gene}\;1)}\times RQ_{\mathrm{sample}\;(\mathrm{reference}\;\mathrm{gene}\;n)}\right)}^{1/n}}$$

The relative expression (RQ) values were determined using the following equation:

$$RQ_{\mathrm{sample}}=E_{\mathrm{gene}}^{\left(Cq_{\mathrm{control}}-Cq_{\mathrm{sample}}\right)}$$

where

*E*_gene_ = Efficiency of the primer pair for the target gene. This efficiency is calculated using the formula (% efficiency × 0.01) + 1, where 100% efficiency = 2.

*Cq*_control_ = Mean Cq value for the control sample.

*Cq*_sample_ = Mean Cq value for each sample containing the target or reference gene.

### 4.10. Statistical Tests

A two-tailed Student’s *t*-test was used to analyze the results obtained from samples with different variances. Statistically different values were considered those with *p* < 0.05.

## 5. Conclusions

Our research group explores how carbon sources regulate antimicrobial synthesis in *Streptomyces*, a genus that produces over 60% of clinically valuable antibiotics. Previous studies have shown that glucose negatively affects four key transcriptional regulators. Among these, we focused on SCO7424 due to its strong glucose-induced overexpression and a broad range of potential target genes. Computational analysis revealed that SCO7424 contains conserved amino acids essential for ligand response and exhibits the characteristic MarR structure. Notably, *sco7424* forms an operon with *sco7425*, which encodes an efflux pump, a common feature among MarR family members. Compared to the wild type, the OVE mutant showed stunted growth, delayed glucose uptake, and lower levels of ACT and RED antibiotics. This decline in antibiotic production was linked to the downregulation of the master regulators actII-ORF4 and redD, positioning the SCO7423 protein as a key regulator that represses secondary metabolite production.

Under repressive conditions, the OVE mutant also overexpressed the neighboring gene *SCO7423,* suggesting that the SCO7423 protein may be part of a two-component system that represses ACT synthesis and shapes morphological development. Since MarR family regulators are well known for their role in antibiotic synthesis in *Streptomyces*, these proteins, directly or indirectly, have a profound impact on secondary metabolite production and cellular differentiation. Understanding the regulatory control mechanisms of this family is crucial for boosting industrial yields of valuable metabolites.

## Figures and Tables

**Table 1:** Growth speed of the different bacterial strains and respective antibiotic production yields.

Bacterial Strain	μ (h^−1^)	Dt (h)	Yx/s (g Biomass/g Glc)	PGP (mg Biomass/mL)	YACT/s YRED/s (g/g Glc)		MPPACT MPPRED (µmol/g Biomass)	
*S. coelicolor* M145 (WT)	0.0211 ± 0.001	32.8 ± 2.14	0.686 ± 0.018	4.6775 ± 0.095	0.003 ± 0.0003	0.0002 ± 4.87866 × 10^−5^	9.22 ± 0.553	1.17 ± 0.207
SpSCO7424 (SE)	0.007 ± 0.001	99.02 ± 23.53	0.406 ± 0.062	3.3675 ± 0.625	0.001 ± 0.0001	0.00002 ± 2.40529 × 10^−6^	5.18 ± 0.209	0.21 ± 0.010

PGP: Peak Growth Point; MPPACT: Maximum production point ACT; MPPRED: Maximum production point RED. Glc: Glucose.

**Table 2:** Changes in gene expression of sco7424, actII-ORF4, and some RED biosynthetic pathway genes.

Repressive Condition (With Glucose) OVE vs. WT Strain			
Gene	Normalized Expression (OVE)	Normalized Expression (WT)	Expression Change (OVE/WT)
*redZ* (*sco5881*)	10.76234 ± 0.60708	0.56775 ± 0.02621	18.95
*redD* (*sco5877*)	0.02128 ± 0.00112	0.20581 ± 0.00854	−9.67
*redL* (*sco5892*)	0.00017 ± 6.6 × 10^−5^	0.00298 ± 0.00012	−17.53
*actII-ORF4* (*sco5085*)	0.57909 ± 0.05157	1.50875 ± 0.01327	−2.60
*sco7423*	0.03138 ± 0.00163	0.01416 ± 0.00111	2.21
*sco7424*	1.53827 ± 0.02869	0.12782 ± 0.00505	12.03
Condition without glucose OVE vs. WT strain			
*redZ* (*sco5881*)	1.93013 ± 0.08816	3.03386 ± 0.03936	−1.57
*redD* (*sco5877*)	0.05323 ± 0.00603	0.23275 ± 0.02604	−4.37
*redL* (*sco5892*)	0.00034 ± 7.2 × 10^−5^	0.00107 ± 0.00028	−3.14
*actII-ORF4* (sco5085)	2.15604 ± 0.67115	33.28908 ± 0.80579	−15.44
*sco7423*	0.00502 ± 0.00016	0.01302 ± 0.00104	−2.59
*sco7424*	0.63700 ± 0.06162	0.15240 ± 0.00471	4.18

## Data Availability

Data are available upon request.

## Supplementary Materials

The following supporting information can be downloaded at: https://www.mdpi.com/article/10.3390/antibiotics15010070/s1, Figure S1: Alignment of the nucleotide sequence of the expected amplification product of the *sco7424* and *sco7425* genes (135 bp) and the sequence obtained after sequencing of the purified PCR product; Table S1: Strains and plasmids used in this work; Table S2: Sequence of oligonucleotides used for PCR, RT_PCR and RT_qPCR. References [42,43,44] are cited in the Supplementary Materials.

## Abbreviations

The following abbreviations are used in this manuscript:

CCR	Carbon catabolite repression
BGCs	Biosynthetic gene clusters
ACT	Actinorhodin
RED	Undecylprodigiosin
CPK	A type I polyketide
Glk	Glucokinase
3D	Three-dimensional
H2	Helix 2
OVE	SCO7424 overexpressed mutant
WT	Wild-type *Streptomyces coelicolor* M145 strain
