# Supplementary material for: Impact of the Transcriptional Regulator SCO7424 Overexpression on Antibiotic Production in Streptomyces coelicolor
Source: Antibiotics (Basel). 2026 Jan 8;15(1):70. doi: 10.3390/antibiotics15010070 (PMC12838073; doi:10.3390/antibiotics15010070)
Supplement: Supplementary file 1 [file antibiotics-15-00070-s001.zip › antibiotics-3932898-supplementary.pdf]

Supplementary material

Impact of the Transcriptional Regulator SCO7424 Overexpression on Antibiotic Production in *Streptomyces coelicolor*

Gladys Vega-Sauceda, Karen Villarreal-Gómez, Beatriz Ruiz-Villafán, Romina Rodríguez-Sanoja and Sergio Sánchez *

Instituto de Investigaciones Biomédicas, Universidad Nacional Autónoma de México (UNAM), Mexico City 04510, Mexico; glamarvega@comunidad.unam.mx (G.V.-S.)
* Correspondence: sersan@biomedicas.unam.mx

Table S1.- Strains and plasmids used in this work

Strains	Characteristics	Reference
Streptomyces coelicolor	M145 SCP1− SCP2−	[42]
Streptomyces coelicolor 702	*S. coelicolor* harboring the plasmid pIJ702	This work
Streptomyces coelicolor **S**pSCO7424	S. coelicolor harboring the plasmid pSCO7424	This work
**Plasmids**		
pIJ702	Derived from pIJ101 with an estimated copy number of approximately 50	John Innes Centre
pSCO7424	pIJ702 with *sco7424* gene	This work

Table S2.- Sequence of oligonucleotides used for PCR, RT_PCR and RT_qPCR

Gene/Plasmid	Oligonucleotide name	Sequence (5'-3'	Size (bp)	Reference
sco7424 (MarR)	Fwd_sco7424rt	AGCAGATGATCCGCTACGTG	159	This work
	Rev_sco7424rt	TGACGAGCTGGGTCATGTTC		
sco7423 (ANTAR-GAF)	Fwd_sco7423	GGTACTCGCAGGACCACAAC	91	This work
	Rev_sco7423	CATCTTTTGTCCGTTGGCTCG		
sco7424/ sco7425	Fwd_7424/25	TCAGGACACCTCGGAGGAAG	135	This work
	Rev_7424/25	GATGCCCATGAACGAGATGAC		
sco5892 (RedL)	Fwd_sco5892	AACTTCGCGGCCTTCAACTA	171	This work
	Rev_sco5892	GGTACCGATGAACCGTGCTA		
sco5085 (ActII-ORF4)	Fwd_actII	AAGGAATATCGCGCACCTGGAAG	102	[43]
	Rev_actII	GTTCCGGAATCATCGGCCCTATTC		
sco5877 (RedD)	Fwd_RedD	GACCTGGTGGACGAACTGTG	99	[44]
	Rev_RedD	ACGCTCGTTGAGCACTTTC		
sco5881 (RedZ)	Fwd_RedZ	TCTTCACCATGGACAGCATCGAC	191	[43]
	Rev_RedZ	GGCATTGACTTCGGTGATGGTTC		
sco5820 (HrdB)	Fwd_hrdB	GCATGCTCTTCCTGGACCTCAT	93	[7]
	Rev_hrdB	TGGAGAACTTGTAGCCCTTGGTGTA		
PIJ702	Fwd_pIJ702	GGCGTCGTAGCGGCCGGTGCGCTTGAG	1467	This work
	Rev_pIJ702	AGCCGGGACTACTGCCG CCGCTCCTGC		

Figure S1.- Alignment of the nucleotide sequence of the expected amplification product of the *sco7424* and *sco7425* genes (135 bp) and the sequence obtained after sequencing of the purified PCR product.

Score	Expect	Identities	Gaps	Strand
250 bits(135)	7e-72	135/135(100%)	0/135(0%)	Plus/Plus

```
Query  1    TCAGGACACCTCGGAGGAAGGCCGCGCATGAGTGCACCCCGTGGAAAGGCCGCCAGTCAG  60
            ||||||||||||||||||||||||||||||||||||||||||||||||||||||||||||
Sbjct  1    TCAGGACACCTCGGAGGAAGGCCGCGCATGAGTGCACCCCGTGGAAAGGCCGCCAGTCAG  60

Query  61   GCCGCCAGTCCCTTCCGGCAGCCGAAGGCCGTCTGGGCGGTCGCCTTCGCCTGCGTCATC  120
            ||||||||||||||||||||||||||||||||||||||||||||||||||||||||||||
Sbjct  61   GCCGCCAGTCCCTTCCGGCAGCCGAAGGCCGTCTGGGCGGTCGCCTTCGCCTGCGTCATC  120

Query  121  TCGTTCATGGGCATC  135
            |||||||||||||||
Sbjct  121  TCGTTCATGGGCATC  135
```
